# MicroRNA-132 attenuated cardiac fibrosis in myocardial infarction-induced heart failure rats

**DOI:** 10.1042/BSR20201696

**Published:** 2020-09-16

**Authors:** Guoyu Wang, Ruzhu Wang, Zhongbao Ruan, Ling Liu, Yong Li, Li Zhu

**Affiliations:** 1 Department of Cardiology, Taizhou People’s Hospital, Taizhou, China; 2 Department of Cardiology, The First Affiliated Hospital with Nanjing Medical University, Nanjing, China

**Keywords:** cardiac dysfunction, cardiac fibroblasts, fibrosis, heart failure, microRNA -132

## Abstract

The aim of the present study was to determine the effect of microRNA (miR)-132 on cardiac fibrosis in myocardial infarction (MI)-induced heart failure and angiotensin (Ang) II-treated cardiac fibroblasts (CFs). Experiments were carried out in Sprague-Dawley rat treatment with ligation of left coronary artery to induce heart failure, and in CFs administration of Ang II to induce fibrosis. The level of miR-132 was increased in the heart of rats with MI-induced heart failure and the Ang II-treated CFs. In MI rats, left ventricle (LV) ejection fraction, fractional shortening, the maximum of the first differentiation of LV pressure (LV +d*p*/d*t*_max_) and decline (LV -d*p*/d*t*_max_) and LV systolic pressure (LVSP) were reduced, and LV end-systolic diameter (LVESD), LV end-diastolic diameter (LVEDD), LV volumes in systole (LVVS) and LV volumes in diastole (LVVD) were increased, which were reversed by miR-132 agomiR but deteriorated by miR-132 antagomiR. The expression levels of collagen I, collagen III, transforming growth factor-β (TGF-β), and α-smooth muscle actin (α-SMA) were increased in the heart of rat with MI-induced heart failure and CFs administration of Ang II. These increases were inhibited by miR-132 agomiR but enhanced by miR-132 antagomiR treatment. MiR-132 inhibited PTEN expression, and attenuated PI3K/Akt signal pathway in CFs. These results indicated that the up-regulation of miR-132 improved the cardiac dysfunction, attenuated cardiac fibrosis in heart failure via inhibiting PTEN expression, and attenuating PI3K/Akt signal pathway. Up-regulation of miR-132 may be a strategy for the treatment of heart failure and cardiac fibrosis.

## Introduction

Heart failure, a complex syndrome resulting from structural or functional cardiac disorders, can disable the ventricle from ejecting or filling blood [1–3]. Despite the progress in diagnosis, heart failure is still a leading cause of morbidity and mortality worldwide [4–6]. Heart failure is preceded by left ventricular (LV) remodeling, which is characterized by the formation of cardiac interstitial fibrosis, including the increases of collagen I, collagen III, α-smooth muscle actin (α-SMA), and transforming growth factor-β (TGF-β) [7–9].

MicroRNAs (miRs), a group of small, naturally occurring and non-coding RNAs, can negatively regulate gene expressions through promoting mRNA degradation or inhibiting mRNA translation [10–13]. Many miRNAs have been recognized as biomarkers and possible therapeutic targets for the diagnosis and treatment of diseases [14]. Human studies and animal experiments have found multiple miRs, including miR-24, -199b, -100, -195, -208, and -133, are dysregulated in heart failure [15–19]. However, the relevant mechanisms are far from being understood.

Circulating miR-132 level was associated with heart failure, and it rose with the severity of heart failure [20]. However, another study found that miR-132 expression was down-regulated in the blood of heart failure patients [21]. Moreover, overexpression of miR-132 dramatically enhanced the antioxidant stress and antiapoptotic ability of H9C2 cells [21]. MiR-132 activated the phosphateidylinositol 3-kinase/protein kinase (PI3K/Akt) signal transduction pathway via inhibiting phosphatase and tensin homolog (PTEN) expression, thus facilitating cardiocyte proliferation and attenuating apoptosis and cardiac fibrosis in rats with doxorubicin-induced dilated cardiomyopathy (DCM) [22]. However, whether miR-132 attenuates heart failure and reduces cardiac fibrosis in MI-induced heart failure is still not well known. The purpose of the present study was to explore the curative effects of miR-132 on cardiac dysfunction and heart fibrosis in rats with MI-induced heart failure.

## Materials and methods

### Animals

The experiments were performed at Animal Core Facility of Nanjing Medical University using male Sprague-Dawley (SD) rats (180-200g, Vital River Biological Co., Ltd, Beijing, China). The rats were kept in a temperature-controlled room on a 12 h light–dark cycle with free access to standard chow and tap water. All procedures were approved by the Experimental Animal Care and Use Committee of Nanjing Medical University (Nanjing, China) in 2017 (No.14051386), and conducted in accordance with the Guide for the Care and Use of Laboratory Animals (NIH publication No. 85-23, revised 1996).

### Myocardial infarction model

The myocardial infarction (MI) in the rat model was induced by coronary artery ligation with sterile techniques as previously reported [23]. Briefly, the rats (180–200 g), anesthetized with sodium pentobarbital (50 mg/kg, i.p.), were randomly subjected to the ligation of the left anterior descending coronary artery or the sham-operated (Sham) groups. The heart was exposed through a left intercostal thoracotomy with the left coronary artery looped by a single nylon suture. Then, the heart was quickly repositioned into the chest. The Sham rats were treated in the same way as the coronary ligation rats except that their left anterior descending coronary arteries were not ligated.

### Angiotensin II rat model

Male SD rats weighing 180–200 g were infused with Ang II (500 ng/kg/min, Sigma, MO, U.S.A.) or saline (solvent control) via osmotic minipumps (model 2004; ALZET, CA, U.S.A.) with an infusion rate of 0.25  μl/h that were surgically placed below the neck for 4 weeks.

### MiRNA-132 agomiR and antagomiR treatment in rats

To determine the effects of miR-132 on MI-induced HF, the rats were injected with miR-132 agomiR (a 2′OME + 5′chol modified miR-132 agonist, 40 mg/kg/day) or antagomiR (a 2′OME+5′chol modified miR-132 inhibitor, 40mg/kg/day) 7 days after MI via tail vein for three consecutive days. The miR-132 agomiR and antagomiR were obtained from RIBOBIO (Guangzhou, China). After 4 weeks of injection, the rats were killed with an overdose of sodium pentobarbital (100 mg/kg, i.p.).

### Echocardiography

Transthoracic echocardiography was performed using an ultrasound system (VisualSonics, Toronto, Canada) with a 21-MHz probe under isoflurane (2.0%) anesthesia. Measurements over three consecutive cardiac cycles were averaged. The ejection fraction (EF) and fractional shortening (FS) of left ventricular (LV) in rats were calculated. The LV end-systolic diameter (LVESD), LV end-diastolic diameter (LVEDD), LV volumes in systole (LVVS), and LV volumes in diastole (LVVD) were measured.

### Hemodynamic monitoring

The rats were anesthetized with isoflurane (2.0%). A conductance micromanometer catheter (1.4F, Millar Instruments, TX, U.S.A.) was inserted into the LV chamber for hemodynamic monitoring via the left carotid artery. The maximum of the first differentiation of LV pressure (LV +d*p*/d*t*_max_) and decline (LV -d*p*/d*t*_max_), LV systolic pressure (LVSP), and LV end-diastolic pressure (LVEDP) were obtained using a PowerLab data acquisition system (AD Instruments, Sydney, Australia).

### Quantitative real time-PCR (qRT-PCR)

The rats were killed with an overdose of pentobarbital (100 mg/kg, i.p.), and the hearts were removed. The total RNA in samples was extracted with TRIzol (Ambion, TX, U.S.A.). The cDNA was extracted from the RNA with reverse transcription using random primers in a total volume of 10 μl according to the instructions of the PrimeScript™ RT Master Mix (TaKaRa Biomedical Technology, Beijing, China). All cDNA was stored at − 80°C before use. Collagen I, collagen III, TGF-β, and α-SMA mRNA were determined with SYBR Green I fluorescence. All samples were amplified in triplicates for 45 cycles in a 384-well plate. The relative gene expression was determined by calculating the values of Δcycle threshold (Δ*C*t) as a relative quantity to the endogenous control. U6 was as a control to miR-132, and GAPDH was as a control to collagen I, collagen III, TGF-β, and α-SMA. The primers (Genscript, Nanjing, China) are shown in Table 1.

**Table 1:** List of utilized primers for qRT-PCR

Gene	Species	Forward primer	Reverse primer
Collagen I	Rat	TCAAGATGGTGGCCGTTAC	CTGCGGATGTTCTCAATCTG
Collagen III	Rat	CGAGATTAAAGCAAGAGGAA	GAGGCTTCTTTACATACCAC
TGF-β,	Rat	CAGGGAGTAAGGGACACGA	ACAGCAGTTAGGAACCCAGAT
α-SMA	Rat	GTCCCAGACATCAGGGAGTAA	TCGGATACTTCAGCGTCAGGA
miR-132	Rat	ACACTCCAGCTGGGTAACA	CTCAACTGGTGTCGTGGA
U6	Rat	GCTTCGGCAGCACATATACTAAAAT	CGCTTCACGAATTTGCGTGTCAT
GAPDH	Rat	GGCACAGTCAAGGCTGAGAATG	ATGGTGGTGAAGACGCCAGTA

Abbreviations: α-SMA, α-smooth muscle actin; GAPDH, glyceraldehyde-3-phosphate dehydrogenase; miR, microRNA; TGF-β, transforming growth factor-β.

### Isolation and culture of cardiac fibroblasts (CFs)

Rat CFs were isolated from SD rats (1–3 days). Briefly, CFs were separated from cardiomyocytes by gravity separation and grown to confluence on 10-cm cell culture dishes with DMEM media including 10% FBS, 1% penicillin and 1% streptomycin (Biochannel Biotechnology Co., Ltd, Nanjing, China) at 37°C in humid air with 5% CO_2_ and 95% O_2_. The second passage CFs was used in the experiments. CFs were incubated with 10^−6^ M [24,25] Ang II (Sigma, MO, U.S.A.) for 24 h to induce the fibrotic phenotype, and treated with miR-132 agomiR or antagomiR according to the manufacturers’ instructions.

### Bioinformatics analysis and dual-luciferase reporter gene assay

Target gene of miR-132 was determined according to previous study [22]. Briefly, endonuclease sites (SpeI and HindIII) were used to insert PTEN into the pMIR-reporter vector, and the mutation sites of complementary sequences of seed sequences were designed on wild-type PTEN (PTEN-WT). After restriction enzyme cutting, the T4 DNA ligase was used to insert the target fragment into the pMIR-reporter vector. The WT and mutant type (MUT) luciferase reporter plasmids with the correct sequence were cotransfected into HEK-293T with miR-132, respectively. After 48 h of transfection, the cells were collected, disrupted, and centrifuged for 5 min to collect the supernatant. The luciferase kit (Beyotime Biotech Co, Ltd., Shanghai, China) was used to determine the relative light unit (RLU) as its instructions

### Statistical analyses

Data are presented as mean ± standard error of the mean (SE). Using GraphPad Prism 6.0 (GraphPad software Inc., CA, U.S.A.), statistical significance among multiple groups was evaluated by one-way analysis of variance (ANOVA) with the Bonferroni post-hoc test. A two-tailed *P*-value <0.05 was considered statistically significant.

## Results

### Expression of miR-132 in the heart of heart failure rats and Ang II-treated CFs

**Figure 1:**
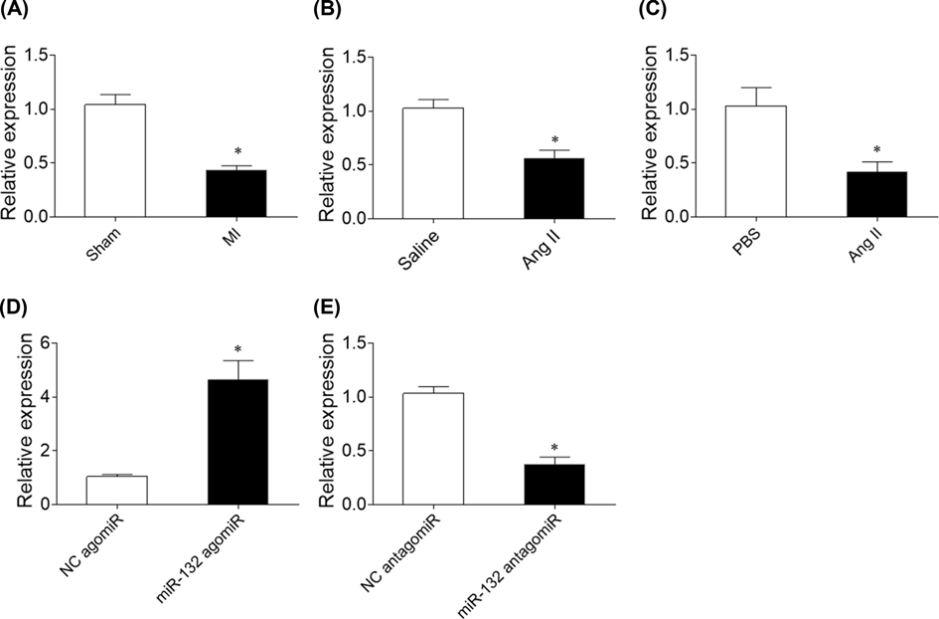
Expression of microRNA (miR)-132 (**A**) The level of miR-132 was reduced in the heart of myocardial infarction (MI)-induced heart failure rats. (**B**) MiR-132 expression level was reduced in the heart of rat administration of angiotensin (Ang) II. (**C**) MiR-132 expression level was reduced in Ang II-treated cardiac fibroblasts (CFs). (**D**) MiR-132 expression level was increased in the heart of rat treatment with miR-132 agomiR. (**E**) MiR-132 expression level was reduced in the heart of rat treatment with miR-132 antagomiR. The results are expressed as mean ± SE; *N* =8; *P<0.05 versus the Sham group (A) or PBS group (B).

The expression level of miR-132 was reduced in the heart of MI-induced heart failure rats (Figure 1A). MiR-132 expression level was reduced in the heart of rat administration of angiotensin (Ang) II (Figure 1B). MiR-132 expression level was reduced in Ang II-treated CFs (Figure 1C) MiR-132 expression level was increased in the heart of rat treatment with miR-132 agomiR (Figure 1D). E. MiR-132 expression level was reduced in the heart of rat treatment with miR-132 antagomiR (Figure 1E).

### Effects of miR-132 agomiR on cardiac function in heart failure rats

**Figure 2:**
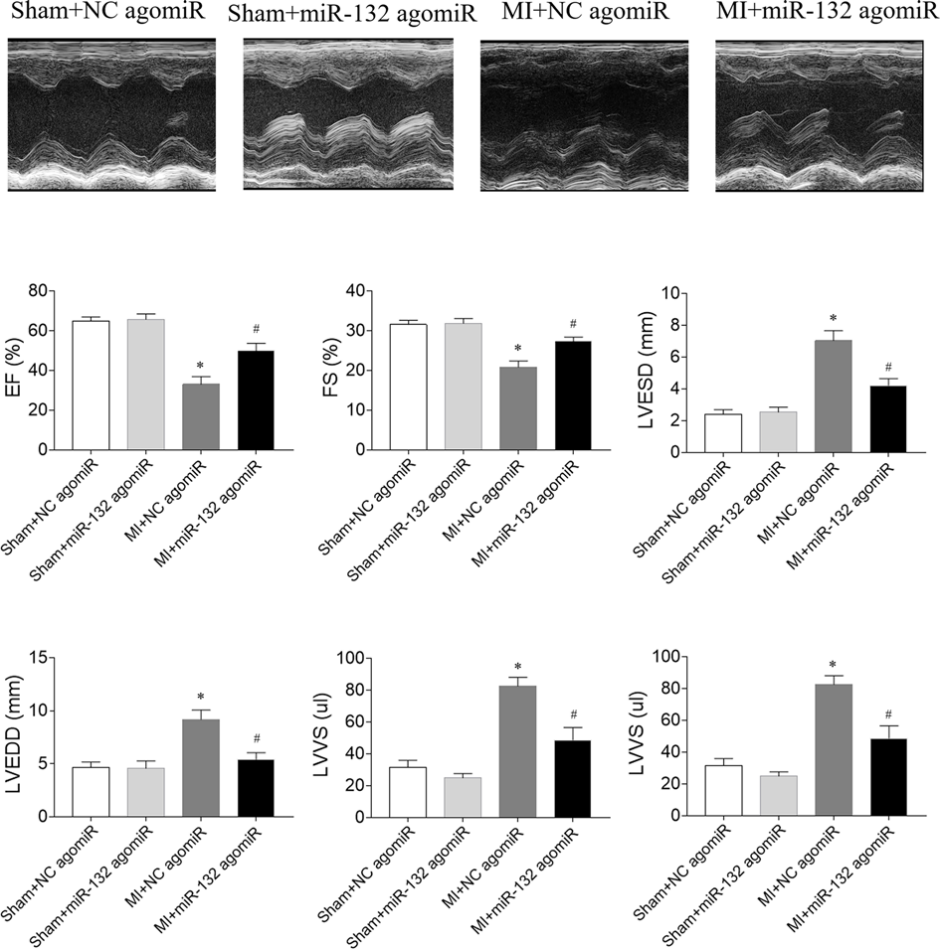
Effects of microRNA (miR)-132 agomiR on cardiac function in myocardial infarction (MI)-induced heart failure rats The left ventricular (LV) ejection fraction (EF) and fractional shortening (FS) were reduced, and LV end-diastolic diameter (LVEDD), LV end-systolic diameter (LVESD), LV volumes in systole diastole (LVVs) and LV volumes in diastole (LVVd) were increased in MI-induced heart failure rats. These changes were reversed by miR-132 agomiR treatment. The results are expressed as mean ± SE; *N*=8; *P<0.05 versus the Sham+NC agomiR group; ^#^*P*<0.05 versus the MI+NC agomiR group.

The EF and FS of LV in MI-induced heart failure rats were reduced, which were improved by miR-132 agomiR. LVESD, LVEDD, LVVS, and LVVD were increased in HF rats, and miR-132 agomiR treatment inhibited these increases (Figure 2).

### Effects of miR-132 antagomiR on cardiac function in heart failure rats

**Figure 3:**
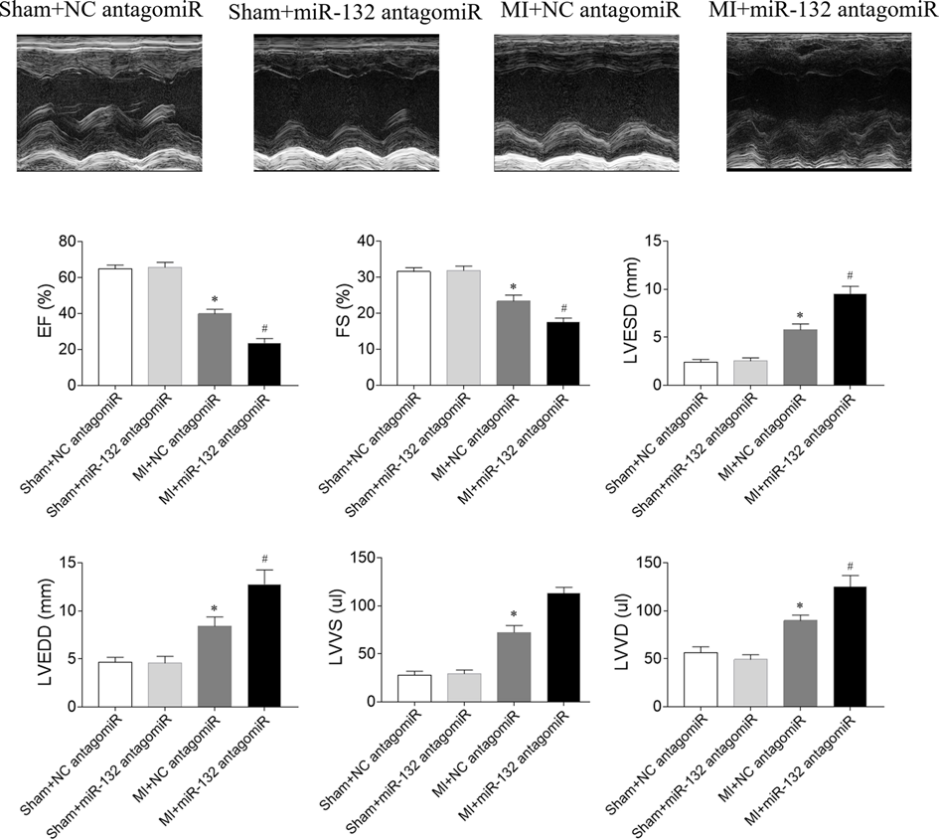
Effects of microRNA (miR)-132 antagomiR on cardiac function in myocardial infarction (MI)-induced heart failure rats The left ventricular (LV) ejection fraction (EF) and fractional shortening (FS) were reduced, and LV end-diastolic diameter (LVEDD), LV end-systolic diameter (LVESD), LV volumes in systole diastole (LVVs) and LV volumes in diastole (LVVd) were increased in MI-induced heart failure rats, and these changes were further aggravated by miR-132 antagomiR treatment. The results are expressed as mean ± SE; *N*=8; *P<0.05 versus the Sham+NC antagomiR group; ^#^*P*<0.05 versus the MI+NC antagomiR group.

The decreases of EF and FS were aggravated by miR-132 antagomiR in MI-induced heart failure rats. The increases of LVESD, LVEDD, LVVS and LVVD in MI rats were further enhanced by miR-132 agomiR injection (Figure 3).

### Effects of miR-132 agomiR on cardiac hemodynamics in heart failure rats

**Figure 4:**
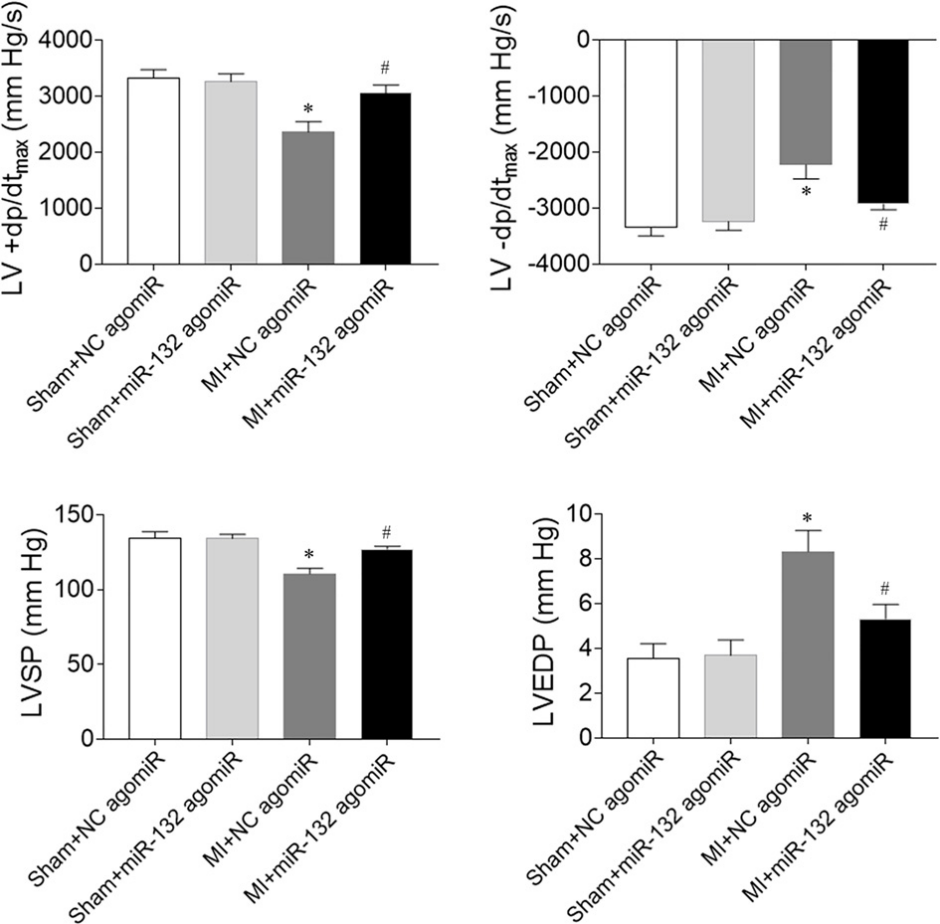
Effects of microRNA (miR)-132 agomiR on cardiac hemodynamics in myocardial infarction (MI)-induced heart failure rats The maximum of the first differentiation of left ventricular pressure (LV +d*p*/d*t*_max_) and decline (LV -d*p*/d*t*_max_) and left ventricle systolic pressure (LVSP) were reduced, and LV end-diastolic pressure (LVEDP) was increased in MI-induced heart failure rats, and these changes were reversed by miR-132 agomiR treatment. The results are expressed as mean ± SE; *N*=8; *P<0.05 versus the Sham+NC agomiR group; ^#^*P*<0.05 versus the MI+NC agomiR group.

MI-induced heart failure reduced LV +d*p*/d*t*_max_ and LV -d*p*/d*t*_max_. MiR-132 agomiR treatment increased the reduction of LV +d*p*/d*t*_max_ and LV -d*p*/d*t*_max_ in heart failure rats. LVSP was reduced in MI-induced heart failure rats, which was reversed after miR-132 agomiR injection. LVEDP in heart failure rats was increased, which was inhibited by miR-132 administration (Figure 4).

### Effects of miR-132 antagomiR on cardiac hemodynamics in heart failure rats

**Figure 5:**
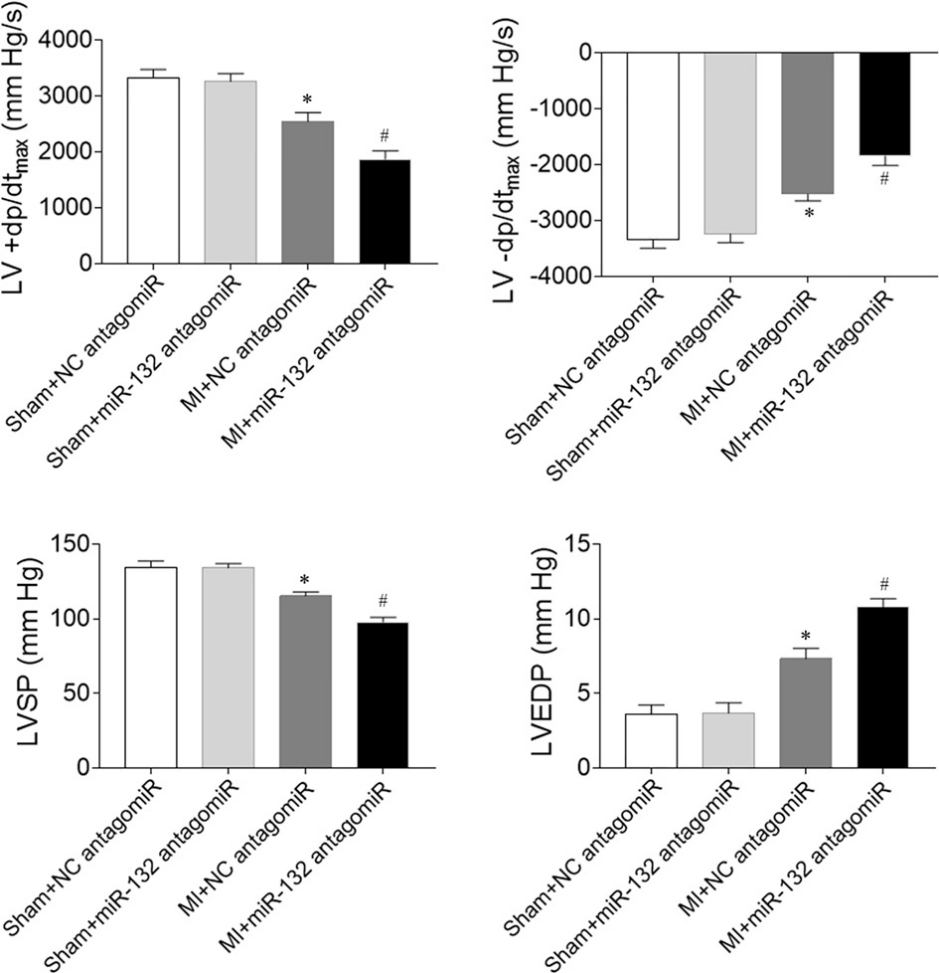
Effects of microRNA (miR)-132 antagomiR on cardiac hemodynamics in myocardial infarction (MI)-induced heart failure rats The maximum of the first differentiation of left ventricular pressure (LV +d*P*/dt_max_) and decline (LV -d*P*/d*t*_max_) and left ventricle systolic pressure (LVSP) were reduced, and LV end-diastolic pressure (LVEDP) was increased in MI-induced heart failure rats, and these changes were further aggravated by miR-132 antagomiR treatment. The results are expressed as mean ± SE; *N*=8; *P<0.05 versus the Sham+NC antagomiR group; ^#^*P*<0.05 versus the MI+NC antagomiR group.

The decreases of LV +d*P*/d*t*_max_ and LV -d*P*/d*t*_max_ were further reduced by miR-132 antagomiR in MI-induced heart failure rats. Treatment with miR-132 antagomiR also further aggravated the decreases of LVSP in MI-induced heart failure rats. The increase of LVEDP in heart failure rats was enhanced after miR-132 antagomiR administration (Figure 5).

### Effects of miR-132 agomiR on cardiac fibrosis in heart failure rats

**Figure 6:**
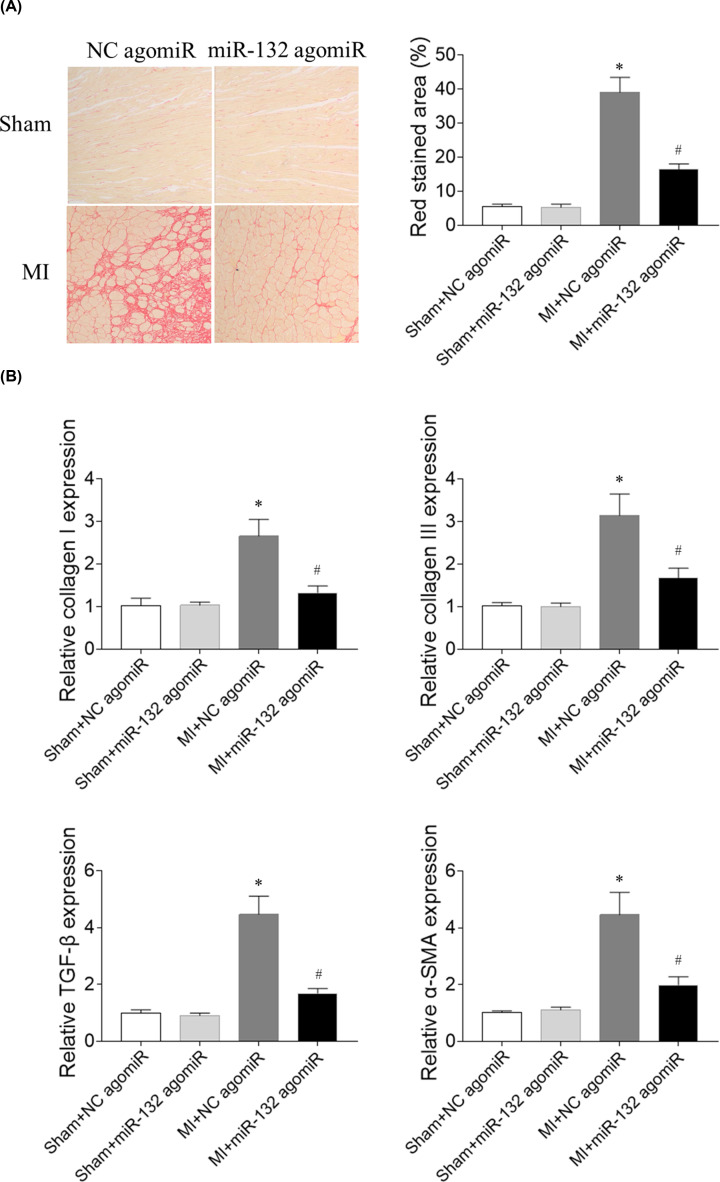
Effects of microRNA (miR)-132 agomiR on cardiac fibrosis in myocardial infarction (MI)-induced heart failure rats (**A**) The increase of fibrosis in heart was inhibited by miR-132 agomiR treatment. (**B**) The expression levels of collagen I, collagen III, transforming growth factor-β (TGF-β), and α-smooth muscle actin (α-SMA) were increased in the heart of MI-induced heart failure rats, and these increases were inhibited by miR-132 agomiR treatment; *N*=8; *P<0.05 versus the Sham+NC agomiR group; ^#^*P*<0.05 versus the MI+NC agomiR group.

The expression level of collagen I was increased in the heart of heart failure rats, which was inhibited after miR-132 agomiR administration. The level of collagen III was increased in the heart of MI rats, which was reversed by miR-132 agomiR treatment. TGF-β level was increased in the heart of MI-induced heart failure rats, which was abolished by miR-132 agomiR injection. The α-SMA expression in the heart was increased in the MI-induced heart failure rats, which was blocked by miR-132 agomiR (Figure 6).

### Effects of miR-132 antagomiR on cardiac fibrosis in heart failure rats

**Figure 7:**
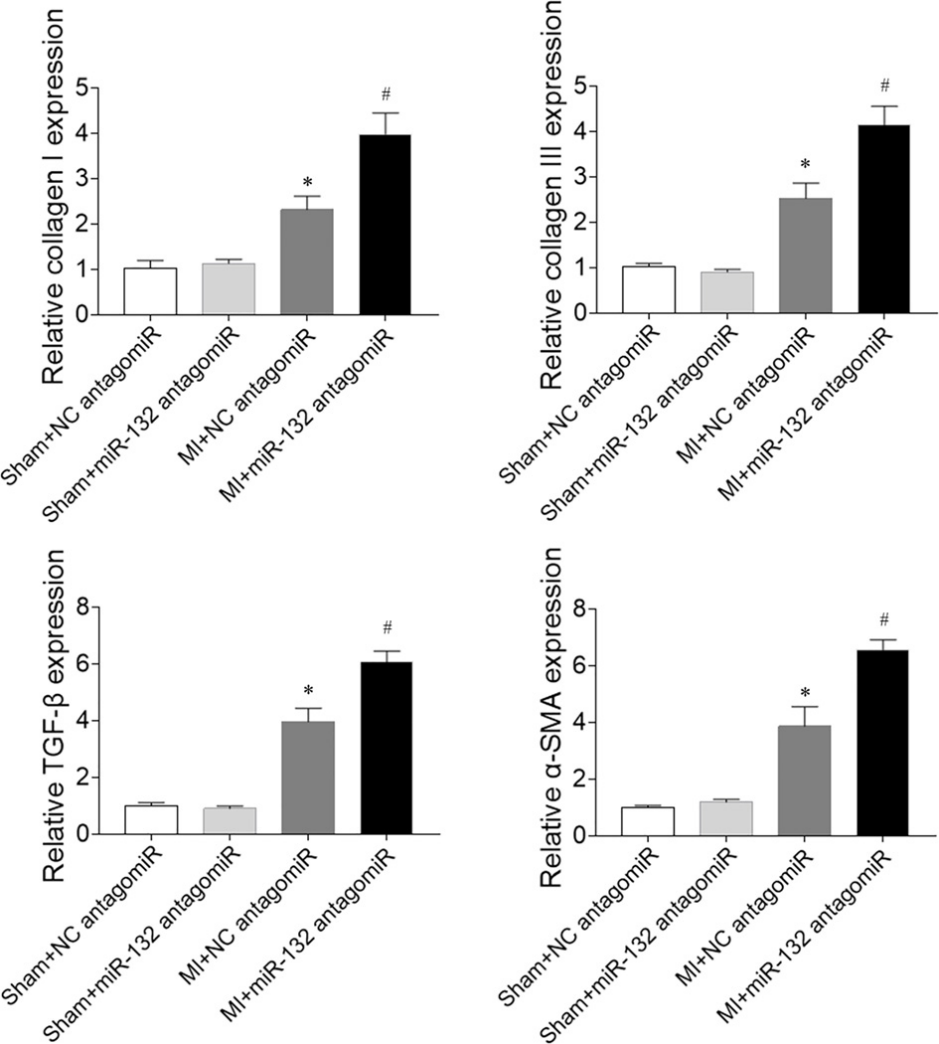
Effects of microRNA (miR)-132 antagomiR on cardiac fibrosis in myocardial infarction (MI)-induced heart failure rats The expression levels of collagen I, collagen III, transforming growth factor-β (TGF-β), and α-smooth muscle actin (α-SMA) were increased in the heart of MI-induced heart failure rats, and these increases were further enhanced by miR-132 antagomiR treatment; *N*=8; *P<0.05 versus the Sham+NC antagomiR group; ^#^*P*<0.05 versus the MI+NC antagomiR group.

The expression levels of collagen I and collagen III in the heart were increased in the MI-induced heart failure rats, and these increases were further enhanced by miR-132 antagomiR administration. MiR-132 antagomiR treatment further elevated the levels of TGF-β and α-SMA compared with NC antagomiR in the heart of MI-induced heart failure rats (Figure 7).

### Effects of miR-132 agomiR on fibrosis in CFs induced by Ang II

**Figure 8:**
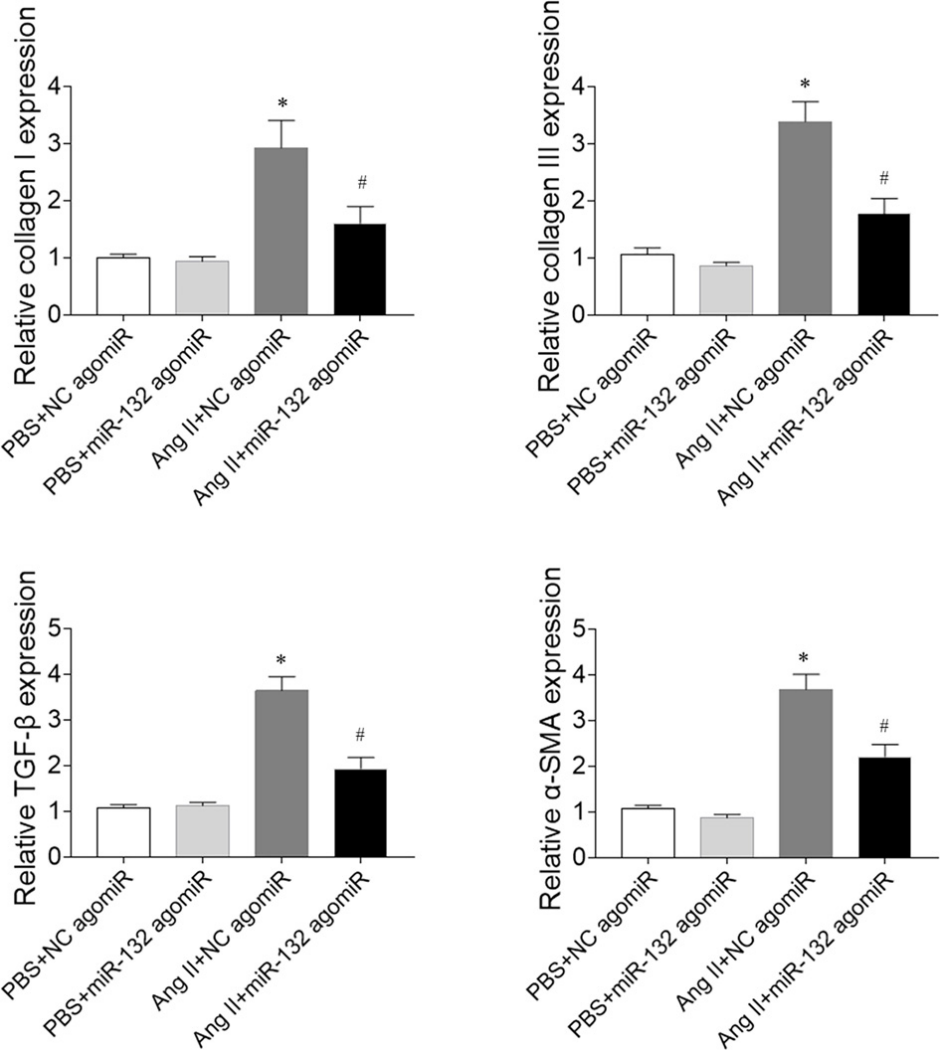
Effects of microRNA (miR)-132 agomiR on fibrosis in cardiac fibroblasts (CFs) treated with angiotensin (Ang) II The expression levels of collagen I, collagen III, transforming growth factor-β (TGF-β), and α-smooth muscle actin (α-SMA) were increased in Ang II-treated CFs, and these increases were inhibited by miR-132 agomiR treatment. *P<0.05 versus the PBS+NC agomiR group; ^#^*P*<0.05 versus the Ang II+NC agomiR group.

The collagen I expression was increased in Ang II-treated CFs, which was inhibited by miR-132 agomiR treatment. The collagen III level was increased in Ang II-treated CFs, which was reversed by miR-132 agomiR administration. TGF-β level was increased in the Ang II-treated CFs, and miR-132 agomiR injection abolished this increase. The α-SMA expression increased in Ang II-treated CFs, and miR-132 agomiR blocked the increase (Figure 8).

### Effects of miR-132 antagomiR on fibrosis in CFs induced by Ang II

**Figure 9:**
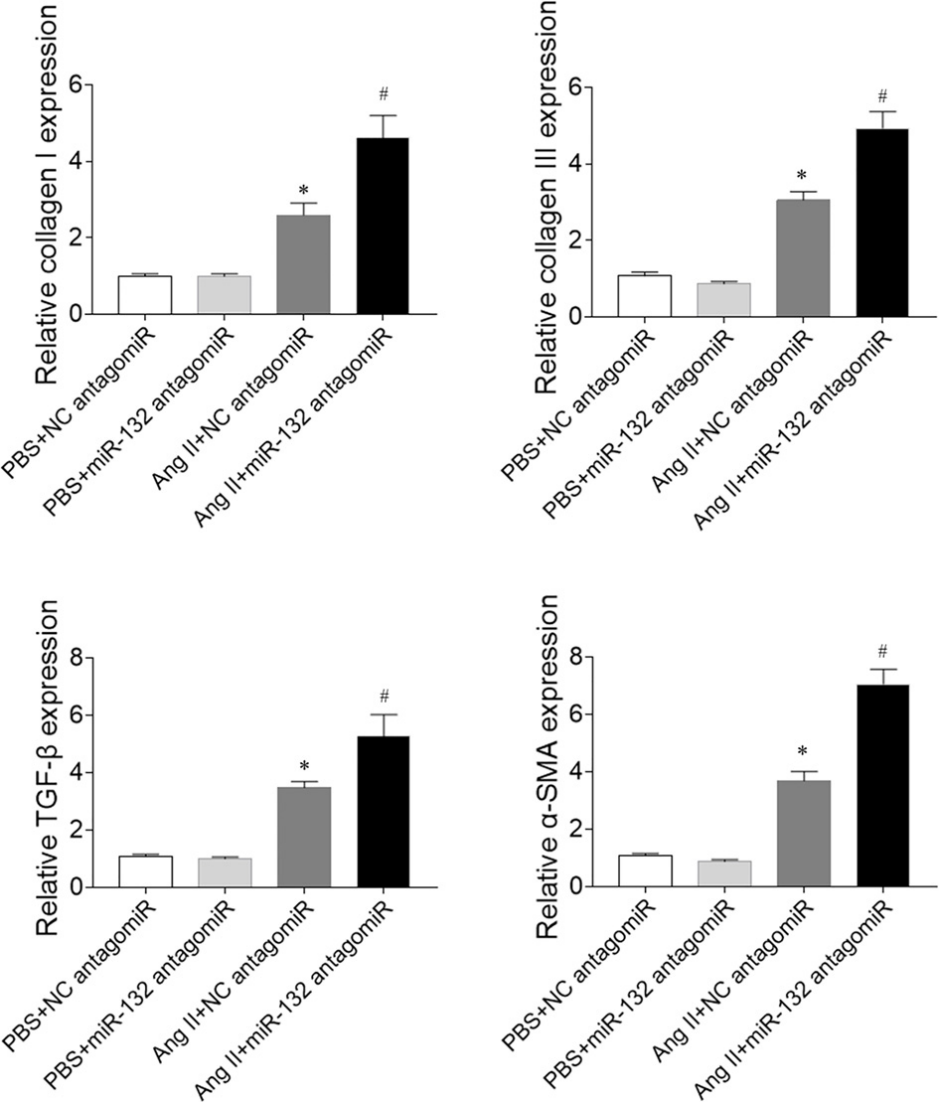
Effects of microRNA (miR)-132 antagomiR on the fibrosis of cardiac fibroblasts (CFs) treated with angiotensin (Ang) II The expression levels of collagen I, collagen III, transforming growth factor-β (TGF-β), and α-smooth muscle actin (α-SMA) were increased in Ang II-treated CFs, and these increases were further enhanced by miR-132 antagomiR treatment. *P<0.05 versus the PBS+NC antagomiR group; ^#^*P*<0.05 versus the Ang II+NC antagomiR group.

The expression levels of collagen I and collagen III in Ang II-treated CFs were increased, which was further enhanced by miR-132 antagomiR administration. The levels of TGF-β and α-SMA in the Ang II-treated CFs were increased, which were also further enhanced by miR-132 antagomiR treatment (Figure 9).

### The mechanism of miR-132

**Figure 10:**
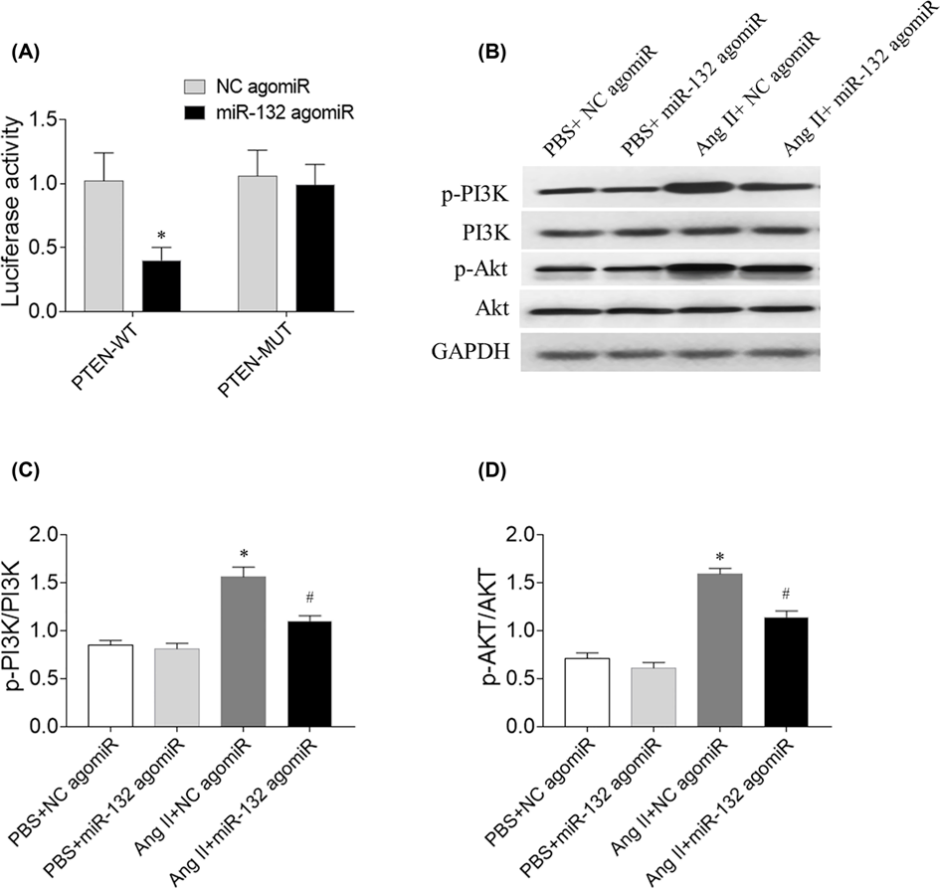
The mechanism of miR-132 (**A**) The luciferase activity of PTEN-WT 3′-UTR was markedly attenuated by miR-132 agimiR. (**B–D**) The increases of p-PI3K and p-Akt were inhibited by miR-132 agomiR. *P<0.05 versus the NC agomiR (**A**) or PBS+NC agomiR (**B**) group; ^#^*P*<0.05 versus the Ang II+NC agomiR group.

Compared with the NC group, the luciferase activity of PTEN-WT 3′-UTR was markedly attenuated by miR-132 agimiR, whereas the PTEN-MUT 3′-UTR luciferase activity was not inhibited (Figure 10A). The levels of p-PI3K and p-Akt were increased in Ang II-treated CFs, and these increases were inhibited by miR-132 agomiR (Figure 10).

## Discussion

In the present study, we found that attenuated cardiac function in MI-induced heart failure rats, which was improved by miR-132 agomiR but aggravated by miR-132 antagomiR treatment. MI-induced heart failure reduced the cardiac hemodynamics, which was reversed by miR-132 agomiR but deteriorated by miR-132 antagomiR administration. MiR-132 agomiR treatment inhibited the cardiac fibrosis in heart failure rats, while miR-132 antagomiR enhanced the fibrosis in the heart of MI rats. Up-regulation of miR-132 with agomiR attenuated the increase of fibrosis in Ang II-treated CFs, but down-regulation of miR-132 with antagomiR further enhanced the increase of fibrosis in Ang II-induced CFs.

The expression of miR-132 was increased in isoproterenol-induced cardiac hypertrophy [26]. MiR-132 expression was down-regulated in the blood of heart failure patients [21]. However, another study showed that circulating miR-132 levels rose with the severity of heart failure in patients with chronic heart failure [20]. In the present study, we found miR-132 expression in the heart was reduced in MI-induced heart failure rats. Furthermore, miR-132 level was also reduced in Ang II-treated CFs. Our current results demonstrated miR-132 level was down-regulated in the heart of heart failure rats and Ang-II treated CFs. Up-regulation of miR-132 may be a therapeutic strategy to treat heart failure and cardiac fibrosis.

The cardiac function was reduced in MI-induced heart failure rats [27], mice [28,29], rabbits [30], and swines [31]. In the present study, the results showed that the EF and FS of LV in MI-induced heart failure rats were reduced, which were improved by miR-132 agomiR. The LVESD, LVEDD, LVVS, and LVVD were increased in the heart failure rats, and miR-132 agomiR treatment inhibited these increases. The decreases of EF and FS, and the increases of LVESD, LVEDD, LVVS and LVVD in MI rats were further enhanced after the administration of miR-132 antagomiR. These results indicated upregulation of miR-132 improved, but downregulation of miR-132 further deteriorated the cardiac dysfunction in heart failure rats, which is supported by the previous finding that injection of miR-132 mimics into MI mice increased FS and EF [32].

Cardiac hemodynamics were impaired in rats with chronic heart failure, which was manifested by the increase of LVEDP and the reduction of LVSP and +dp/dt_max_ [23]. In the present study, we found that miR-132 agomiR treatment enhanced the reduction of LV +d*P*/d*t*_max_, LV -d*P*/dt_max_ and LVSP, and attenuated the increase of LVEDP in heart failure rats. The decreases of LV +d*P*/d*t*_max_, LV -d*P*/d*t*_max_ and LVSP, and the increase of LVEDP was further aggravated by miR-132 antagomiR administration. These results demonstrated that the inhibition of miR-132 further damaged the cardiac hemodynamics in heart failure rats while increasing the miR-132 level improved cardiac hemodynamics.

Cardiac fibrosis is caused by pathological stimulation to the heart. MI-induced heart failure showed markedly fibrosis in the heart [33]. The Ang II-induced activation of cardiac fibroblasts, and hypertrophy and proliferation of cardiomyocytes were significantly inhibited by miR-132 inhibitor anti-miR-132 [34]. Up-regulated miR-132 facilitated cardiocyte proliferation and repressed cardiocyte apoptosis and cardiac fibrosis in dilated cardiomyopathy induced by doxorubicin [22]. In the present study, the results showed that the expression levels of collagen I, collagen III, TGF-β, and α-SMA were increased in the heart of heart failure rats, which was reversed by miR-132 agomiR treatment, but further enhanced by miR-132 antagomiR injection. Moreover, the expression levels of collagen I, collagen III, TGF-β, and α-SMA in Ang II-treated CFs were inhibited by miR-132 agomiR treatment and further increased by miR-132 antagomiR treatment. These results indicated that miR-132 up-regulation attenuated the fibrosis of heart and inhibited the activation of cardiac fibroblasts in MI rats.

Differences genes are targeted by miR-132 in some diseases as previous studies. It showed that miR-132 inhibits cardiomyocyte apoptosis, and ameliorates myocardial remodeling in rats with MI through IL-1β down-regulation [35]. miR-132 exhibited the protective impacts on H9C2 cells against oxygen and glucose deprivation-induced injury via targeting FOXO3A [36]. Our present study found that miR-132 targeted PTEN in CFs, which is supported by previous study [22]. The signal pathway of PI3K/Akt was involved in the fibrosis of the heart [37,38]. The expression of p-Akt was increased in the CFs treated by Ang II [8]. In our present study, the results showed that p-PI3K and p-Akt levels were increased in Ang II-treated CFs, and these results indicated that miR-132 inhibited PTEN expression, and attenuated PI3K/Akt signal pathway in CFs.

In conclusions, up-regulation of miR-132 improved the cardiac dysfunction and the damage to cardiac hemodynamics, and attenuated cardiac fibrosis in heart failure via inhibiting PTEN expression, and attenuating PI3K/Akt signal pathway. MiR-132 may help to improve heart failure and is a potential biomarker for heart failure [39].

## Abbreviations

Ang: angiotensin
CF: cardiac fibroblast
CTGF: connective tissue growth factor
EF: ejection fraction
FS: fractional shortening
LV: left ventricle
LV +d*P*/d*t*_max_: the maximum of the first differentiation of LV pressure
LVEDD: LV end-diastolic diameter
LVESD: LV end-systolic diameter
LVSP: LV systolic pressure
LVVD: LV volumes in diastole
LVVS: LV volumes in systole
MI: myocardial infarction
miR: microRNA
SD: Sprague-Dawley
SMA: smooth muscle actin
TGF: transforming growth factor
